# Functional insights into the testis transcriptome of the edible sea urchin *Loxechinus albus*

**DOI:** 10.1038/srep36516

**Published:** 2016-11-02

**Authors:** Juan Diego Gaitán-Espitia, Roland Sánchez, Paulina Bruning, Leyla Cárdenas

**Affiliations:** 1 Instituto de Ciencias Ambientales y Evolutivas, Universidad Austral de Chile, Casilla 567 Valdivia, Chile; 2 CSIRO Oceans & Atmosphere, GPO Box 1538, Hobart 7001, TAS, Australia

## Abstract

The edible sea urchin *Loxechinus albus* (Molina, 1782) is a keystone species in the littoral benthic systems of the Pacific coast of South America. The international demand for high-quality gonads of this echinoderm has led to an extensive exploitation and decline of its natural populations. Consequently, a more thorough understanding of *L. albus* gonad development and gametogenesis could provide valuable resources for aquaculture applications, management, conservation and studies about the evolution of functional and structural pathways that underlie the reproductive toolkit of marine invertebrates. Using a high-throughput sequencing technology, we explored the male gonad transcriptome of this highly fecund sea urchin. Through a *de novo* assembly approach we obtained 42,530 transcripts of which 15,544 (36.6%) had significant alignments to known proteins in public databases. From these transcripts, approximately 73% were functionally annotated allowing the identification of several candidate genes that are likely to play a central role in developmental processes, nutrient reservoir activity, sexual reproduction, gamete generation, meiosis, sex differentiation, sperm motility, male courtship behavior and fertilization. Additionally, comparisons with the male gonad transcriptomes of other echinoderms revealed several conserved orthologous genes, suggesting that similar functional and structural pathways underlie the reproductive development in this group and other marine invertebrates.

Echinoderms and, in particular, sea urchins, play an important ecological role in coastal ecosystems as a food source for higher animals and as a regulators of seagrass and rocky reef communities^1^. These calcifying marine invertebrates are now a focus of intense research as over-fishing of sea urchins for human consumption has reduced their natural populations worldwide^2^. The roe (gonad) is the only edible part of the sea urchin. It is considered a high quality seafood product and its price is greatly influenced by factors such as appearance, colour, texture, and flavour^3^. Beyond their economic and ecological value, sea urchins are also an important model system for climate change biology due to the critical effects induced by ocean warming and acidification on gonad development and reproduction^4^, and also on the metamorphosis and growth of early ontogenetic stages^5^. Like other marine invertebrates, most sea urchins are broadcast spawners, releasing their gametes into the water column, where fertilization occurs^2^. The quality of the gametes and therefore the successful fertilization in these organisms, depend, in part, on the quality of the gonads and their amino acid and carotenoid composition^6^.

Sea urchins have five gonads attached to the inner wall of their exoskeletons. Each gonad consists of hundreds of acini (i.e., clusters of cells) with populations of germ cells and somatic cells called nutritive phagocytes^7^. The dynamic interactions between the germinal and the somatic cellular populations in the gonads, are fundamental processes during gametogenesis of sea urchins^8^. For instance, the nutritive phagocytes provide a structural and nutritional microenvironment for germinal cells^9^ having similar functions to the Sertoli cells of vertebrates^10^. Despite the pivotal role of nutritive phagocytes in the sea urchin gametogenesis, the cellular and molecular mechanisms involved in gonial cell mitosis as well as in the phagocytic and nutritional functions are not fully understood^8^. Some progress has been made in the understanding of the molecular and functional processes underlying gametogenesis in female sea urchins^8^,^11^,^12^. During oogenesis, oocytes express a unique set of genes that appear essential for their growth, for meiotic recombination and division, for storage of nutrients, and for fertilization^11^. In addition, many transcriptional factors have been identified as responsible for the unique transcriptional activity seen in female gametocytes, such as those of the forkhead family (e.g., FoxL2, FoxX, FoxQ2 and FoxN1/4), the ETS factor Sp-Eyg, the doublesex transcription factor dsx, and other regulatory proteins that are involved in ovary and oocyte development, and also in sex determination of different metazoans^11^,^12^,^13^. In contrast, similar genomic and expression analyses are unavailable for sea urchin spermatogenesis. Related processes like formation of the synaptonemal complex and meiosis also occur during spermatogenesis and are probably governed by the same suite of genes observed during oogenesis^8^. However, we are still no closer in understanding the male reproductive toolkit for this highly fecund animal^14^.

One of the major obstacles in defining the regulatory and molecular mechanisms underlying reproductive development of sea urchins is the limited genetic and genomic information available for these organisms. In this sense, transcriptome sequencing is an effective way to discover genes participating in specific biological processes when genome sequence is not available^15^. In this study, we describe the male gonad transcriptome of the Chilean edible sea urchin *Loxechinus albus* (Molina, 1782). This sea urchin is one of the most economically important species in the littoral benthic systems of the southeast Pacific of South America^16^. Harvesting of *L. albus* represents the largest extraction volume among world urchin fisheries^17^. However, overfishing of *L. albus* along Chilean coasts is driving the decline or depletion of natural populations of this biological resource^18^. A more thorough understanding of sea urchin gametogenesis and gonad development could allow the manipulation of these processes at several stages in order to accomplish particular goals such as: i) To suppress gametogenesis in order to produce high-quality gonads for consumption; and ii) to promote gametogenesis for the increased production of seed stock and natural populations recovery^19^.

## Results

### Sequencing and *de novo* assembly of the *L. albus* transcriptome

**Figure 1:**
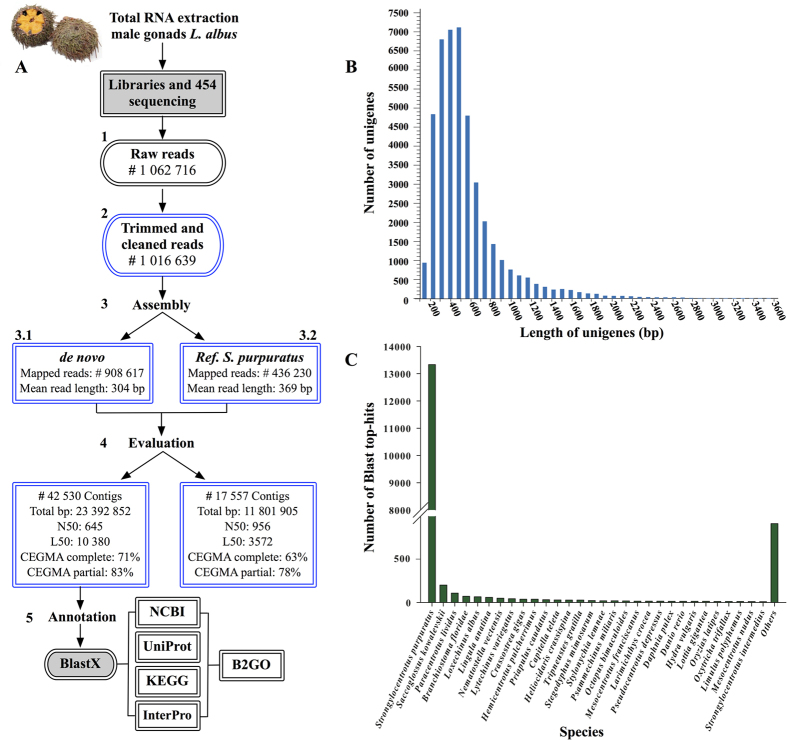
(**A**) Schematic flowchart shows the molecular biology and bioinformatic methods employed in this study. N50 is defined as the length for which the collection of all contigs of that length or longer covers at least half an assembly. L50 is defined as the number of contigs equal to or longer than N50. (**B**) Length distribution of unigenes. (**C**) Top BLASTx hit species distribution obtained against the NCBI non-redundant (nr) protein database.

Sequencing of the male gonads of *L. albus* produced 1,062,716 raw Roche 454 GS-FLX Titanium reads that were quality and adapter trimmed and size selected to yield 1,016,639 cleaned reads with a mean length of 309.8 bp and 295 Mbp of total sequence data (Fig. 1A). The SRA raw reads have been deposited on GenBank public database under the accession number SRP066399 of the bioproject PRJNA302689. After quality control steps, reads were assembled using the *de novo* and the Reference Annotation Based Transcript (RABT) methods (Fig. 1A). Overall, assembly results obtained from RABT with the genome of *S. purpuratus* gave lower mapped reads and number of contigs compared to the *de novo* approach (Fig. 1A). For RABT, assembly of high quality reads generated 17,557 tentative consensus sequences (non-redundant sequences or unigenes), with an N50 of 956 bp and an L50 of 3,572 (Fig. 1A, Table S1). On the other hand, the *de novo* assembly generated 42,530 consensus sequences with an N50 of 645 bp and an L50 of 10,380 (Fig. 1A, Table 1). Further analyses of these two assembled transcriptomes revealed a lower coverage of homologs in UniRef90 for RABT (11 536) in comparison to the *de novo* assembly (15,072) (Supplementary Table S2). Similarly, the assessment of transcriptome completeness (CEGMA) showed a higher representation of the core eukaryotic genes (CEGs) in the *de novo* transcriptome compared to the RABT (Fig. 1A, Table 1). Based on these results, the *de novo* assembly was chosen for all downstream analyses.

Unigenes in our *de novo* assembled transcriptome of *L. albus*, had a mean length of 539 bp (Fig. 1B) with a sequencing coverage ranging from 1 to 4,459 with a mean of ~7 (highly left-skewed toward low coverage).As expected for a randomly fragmented transcriptome, there was a positive relationship between the length of a given unigene and the number of reads assembled into it (Supplementary Fig. S1). The CEGMA analysis of our *de novo* assembly identified 176 out of the 248 core proteins (71%) as complete (defined as >70% alignment length with core protein) and 205 (83%) as partially present (Fig. 1A, Table 1). The average number of orthologs per CEG was 2.11 with the higher percentages in the more divergent groups (i.e., 1 and 2) whilst the total number of CEGs was higher in the highly conserved groups (i.e., 3 and 4) (Supplementary Table S1).

### Functional annotation

Of the 42,530 assembled unigene sequences, 15,544 (36.6%) had significant alignments (≤1e−5) to known proteins in the public databases UniProt (Swiss-Prot and TrEMBL) and NCBI (nr) (Supplementary Table S2), while 26,986 (63.4%) showed no or poor similarity matches and may represent specific unigenes of *L. albus* with unknown function. Our results showed that matching efficiency (i.e., sequences with hits) increases with the length of the unigene sequences (Supplementary Fig. S2). From the unigenes with significant blast hits, 13,333 (~86%) matched to the sea urchin *S. purpuratus*, followed by the acorn worm *Saccoglossus kowalevskii* (~1%) that is a closely related species to echinoderms, and the sea urchin *Paracentrotus lividus* (<1%) (Fig. 1C). Blast results were used for functional categorization of the assembled unigenes. For this step, Blast2GO annotations (52,725 GO terms for 8,144 sequences) were merged with the InterProScan annotations (18,661 GO terms for 8,022 sequences) to maximize the number of sequences with assigned functions. In total, 58,957 GO terms were identified for 11,400 annotated sequences under the three main ontologies. From these sequences, 8,811 (78%) were assigned to molecular functions (GO:0003674), followed by 7,651 (67%) to biological processes (GO:0008150) and 6,425 (56%) to cellular components (GO:0005575) (Supplementary Fig. S3). Within the molecular function category, binding (GO:0005488) and catalytic activity (GO:0003824) were the most represented GO terms with 74% (6,559 sequences) and 48% (4,302 sequences), respectively. For the biological processes, the three top GO terms were cellular (GO:0009987, 81%), metabolic (GO:008152, 73%) and single-organism (GO:0044699, 66%) processes. Furthermore, in the cellular components category, the predominant GO terms were grouped in cell (GO:0005623, 86%), cell part (GO:0044464, 85%) and organelle (GO:0043226, 69%).

**Figure 2:**
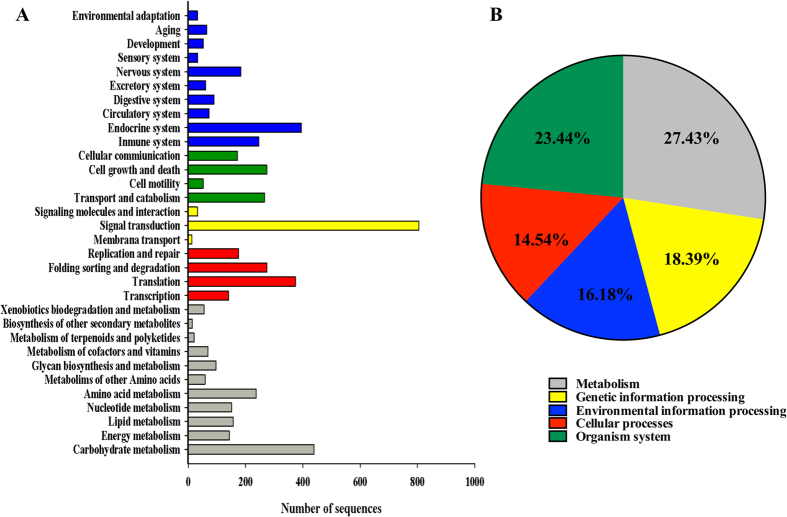
Unigenes homology to KEGG genes obtained from the KAAS server. (**A**) Number of sequences assigned to each sub-category of the reference hierarchy KOs. (**B**) Percentage distribution of the five top KEGG orthology categories in the male gonad transcriptome of *Loxechinus albus*.

**Figure 3:**
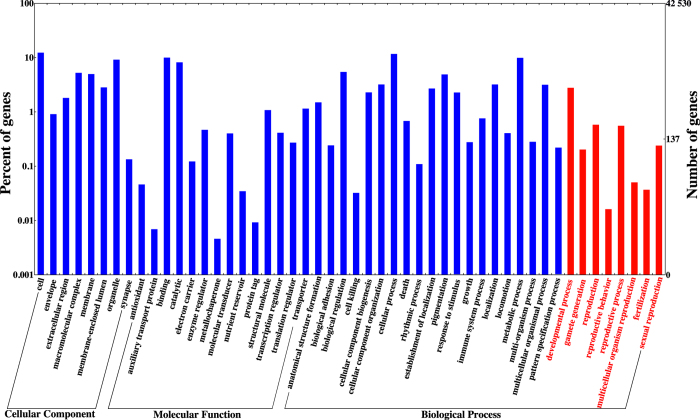
Distribution of Gene Ontology (GO) assignments of assembled unigenes of *L. albus*. GO categories are shown on the x-axis grouped into three main categories: biological processes, cellular components and molecular functions. The y-axis indicates the percentage of total genes in each category.

In order to identify functional pathways active in male gonads of *L. albus*, all unigenes were mapped to the reference canonical pathways in KEGG database. In total, 7,067 unigenes were mapped to 446 KEGG pathways within 48 categories (Supporting information). From these, 5,252 unigenes were assigned to the main KO categories (Fig. 2A) with a major representation of Metabolism and Organism System (50.87%), followed by Genetic Information Processing, Environmental Information Processing and Cellular Processes (49.12%) (Fig. 2B). Within these five categories, the highest represented pathways included signal transduction (15.32%), carbohydrate metabolism (8.36%), endocrine system (7.52%), translation (7.12%), folding sorting and degradation (5.24%), cell growth and death (5.22%) (Fig. 2A). The GO and KEGG annotations were helpful for identifying potential genes related to developmental processes (1204; GO:0032502), reproduction (252; GO:0000003), reproductive processes (214; GO:0022414), sexual reproduction (104; GO:0019953), gamete generation (88; GO:0007276), multicellular organism reproduction (22; GO:0032504), fertilization (16; GO:0009566) and reproductive behaviour (7; GO:0019098) (Fig. 3, Supplementary Table S3). Interestingly, our GO analysis revealed transcripts related to specific process such as spermatogenesis (GO:0007283), spermatid development (GO:0007286), male meiosis (GO:0007140), regulation of meiosis (GO:0040020), sperm ejaculation (GO:0042713), male courtship behavior (GO:0008049), sex differentiation (GO:0007548), sperm and flagellar motility (GO:0030317 and GO:0001539), binding of sperm to zona pellucida (GO:0035036) and fusion of sperm to egg plasma membrane (GO:0007339) (Supplementary Table S3). Furthermore, in terms of reproductive development, our analysis uncovered genes related to male gonad development (GO:0008584), genitalia morphogenesis (GO:0035112), pigmentation during development (GO:0048066), Leydig (GO:0033327) and Sertoli (i.e., the nutritive phagocytes) cell differentiation (GO:0060008) (Supplementary Table S3). The nutritional role of the Sertoli cells may explain the identification of genes associated to nutrient reservoir activity (GO:0045735) in testis of *L. albus* (Supplementary Table S3).

### Orthologous clusters in male gonads of echinoderms

**Figure 4:**
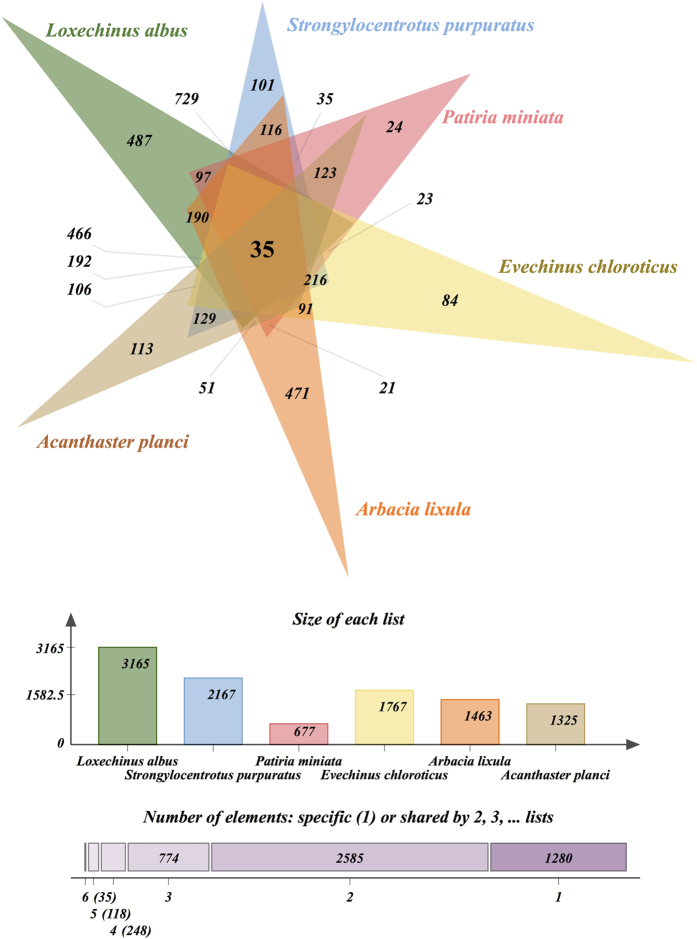
Comparisons of orthologous clusters among male gonad transcriptomes of the sea urchins *Loxechinus albus, Arbacia lixula, Evechinus chloroticus, Strongylocentrotus purpuratus*, and the sea stars *Patiria miniata* and *Acanthaster planci*.

Comparisons with the testis transcriptomes of other sea urchins and sea stars revealed a total of 5,040 clusters from which 3,760 orthologous clusters contains at least two species. Overall, 35 clusters were shared among all the six species (Fig. 4). These clusters involve proteins related to cell division and growth processes (e.g., Transitional endoplasmic reticulum ATPase E), ATP binding and microtubule motor activity (e.g., Kinesin-like protein FLA10), rotation of flagellar microtubules (e.g., Kinesin-like protein KLP1), cell cycle progression and developmental events (e.g., Histone deacetylase HDT3), mitochondrial (e.g., Cytochrome c oxidase subunit 3) and catalytic (e.g., Isovaleryl-CoA dehydrogenase) activity (Supplementary Table S4 and S5). Posterior analyses of GO terms revealed a significant GO enrichment of the cellular component kinesin complex (GO:0005871), and the molecular functions hydrolase activity (GO:0016787) and microtubule motor activity (GO:0003777). Comparisons between *L. albus* and the testis transcriptome of its closest available phylogenetic relative (i.e., sharing the most common recent ancestor), the black sea urchin *A. lixula*, revealed 190 orthologous clusters (Fig. 4), in which most of the proteins were grouped into the GO terms anterior/posterior axis specification (GO:0009948), establishment of planar polarity (GO:0001736), proton-transporting V-type ATPase (GO:0033180), translation elongation factor activity (GO:0003746), spermatogenesis (GO:0007283), sperm individualization (GO:0007291) and spermatid development (GO:0007286) (Supplementary Tables S4 and S5). On the other hand, transcriptomic comparisons with the most distant available phylogenetic sea urchin, *S. purpuratus*, revealed 729 clusters of orthologous proteins (Fig. 4), with over-representation of metabolic process (GO:0008152) and multicellular organismal development (GO:0007275) (Supplementary Tables S4 and S5).

## Discussion

The use of sea urchins as a model system to address major paradigms in development and evolution has been strengthened in recent years by the generation of extensive genomic resources, especially for model species such as *S. purpuratus, Lytechinus variegatus, Eucidaris tribuloides* and *Paracentrotus lividus*^20^. Most genomic and transcriptomic studies have been focused on gene regulatory networks during early development e.g. ref. 21, and in a lower proportion on sea urchin gametogenesis, with major progress in oogenesis^8^,^11^,^12^. In order to obtain a more comprehensive understanding of the functional elements and regulatory mechanisms underlying reproductive development and spermatogenesis of sea urchins, we sequenced the male gonad transcriptome of the Chilean edible sea urchin *Loxechinus albus*. In this study, both the *de novo* and the Reference Annotation Based Transcript (RABT) methods were employed to generate the assembled transcriptome of the Chilean sea urchin. Taking advantage of the well-characterized and annotated genome of *S. purpuratus*^22^, we expected to obtain higher quality and completeness for our assembled transcriptome with the RABT approach compared to the *de novo* method. However, contrary to our expectations, our *de novo* assembly resulted in many more mapped reads, assembled transcripts and conserved eukaryotic core genes than with the RABT approach. The lower performance of the RABT could be explained by the genetic divergence between the reference genome of *S. purpuratus* and our target species^15^,^23^, where the estimated divergence time for the split between these two species is dated approximately 55.5 MYA^24^. In this sense, it has been suggested that the use of a reference genome that is too genetically distant from the target species can yield inaccurate read alignments and substantial data loss, decreasing the ability to detect biological variants, isoforms and exogenous transcripts^25^. On the other side of the coin, attempting to assemble a transcriptome from short sequences without a reference genome is a computationally challenging task. Some of the issues faced are related to the substantial memory and long computing times required, the existence of large number of reads with artefacts, sequencing errors and different sequencing coverage among different transcripts^26^, and also the presence of paralogous genes^27^. In this study we selected the CLC *de novo* assembler as it has performed well in a number of previous transcriptome assemblies^28^,^29^,^30^ and it has a faster computing pace with comparable or better assembly results than other bioinformatics programs^31^.

Our *de novo* assembled transcriptome showed similar characteristics to the available transcriptomes of other echinoderms. For instance, the GC content of the testis transcriptome of *L. albus* (40.4%) was closer to the testis (40.9%) and ovary (42.1%) transcriptomes of the black sea urchin *Arbacia lixula*^32^, and showed slightly higher values in relation to the transcriptomes of the Antarctic sea urchin *Sterechinus neumayeri* (38.6%)^15^, the purple sea urchin, *S. purpuratus* (36.9%)^33^, and the kina sea urchin *Evechinus chloroticus* (39%)^34^. Moreover, in terms of the number of assembled unigene sequences, our *de novo* assembled transcriptome was within the range reported in other 454 sequencing projects of sea urchins^15^, sea cucumbers^35^, and sea stars^36^. Although the percentage of unigenes of *L. albus* with a BLAST-hit may appear to be a relatively small number, we found that this value does not differ from other *de novo* transcriptome studies with non-model echinoderms^14^,^15^,^34^,^37^. In this sense, the lack of homology with known proteins could be explained by biological reasons such as: i) the lack of annotated protein sequences of phylogenetically close species; ii) sequences from untranslated regions (UTR) of mRNA; iii) long noncoding RNA; and iv) orphan proteins^38^. These results are congruent to those reported for the transcriptome of the sea urchin *S. intermedius*^39^, the sea star *Acanthaster planci*^14^ and the brittle star *Amphiura filiformis*^40^.

### Genes involved in the regulation of gonadal development and gamete production

In echinoderms, gametogenesis and gonad development are prolonged processes of interactions between germinal and somatic cells^8^,^41^. These processes involve cell growth, new organelle synthesis and storage, cell differentiation, sex determination, meiosis and the activity of a set of genes related to reproduction, growth and metabolism^12^,^14^,^36^,^41^. By combining results from our different functional analyses we were able to identify several genes that are specifically related to these processes in male sea urchins. For instance, genes such as the DNA helicase MCM8, the Chd5 (chromodomain-helicase-DNA-binding protein Mi-2), the Yy1 (transcriptional repressor protein YY1), the Sfmbt1 (Scm-like with four MBT domains protein 1) and the Nphp1 (Nephrocystin-1) have been linked to different stages of spermatid differentiation^42^ and male gamete generation^43^,^44^,^45^. Moreover, genes such as the Dmrt1 (Doublesex- and mab-3-related transcription factor 1), the Rara (Retinoic acid receptor alpha) and the TMF1 (TATA element modulatory factor) have been recognized to play a role during testis development^46^, male germ cell proliferation and differentiation of Sertoli and Leydig cells^47^. These two types of somatic cells are fundamental in spermatophore formation, gamete production and epididymal sperm maturation by regulating the synthesis of testosterone and growth factors^48^ and by providing a structural and nutritional microenvironment for germinal cells^9^. Other key genes involved in spermatogenesis belong to the serine/threonine protein kinase complexes (e.g., TSSK4, VRK1, HASPIN, MAK), which are composed of a regulatory subunit, cyclin and a catalytic subunit, cyclin-dependent kinase (Cdk), that support germ cell maturation^14^.

Our functional analyses also found several signalling pathways that have been documented to be essential in spermatozoa and gonadal processes. For instance, signaling pathways of the GTPases Rap1 (KO04015) and Ras (KO04014) have been associated to spermatogenesis and sperm motility of sea urchins and vertebrates^49^,^50^. Furthermore, signalling pathways of some protein kinases have been related to capacitation, acrosome reaction and phosphorylation of sperm heads (MAPK: KO04010), energy homeostasis of spermatozoa (AMPK: KO04152) and sperm-oocyte interaction (ErbB: KO04012)^48^. Within these pathways, some kinase receptors (e.g., RTK) bind a variety of growth factors (e.g., TGF-β: KO04350; VEGFR: KO04370), mediating cell-cell interactions during sea urchin development^51^. Other signalling pathways that are known to be involved in a variety of biological processes, including development, are the wnt (KO04310), the Hedgehog (KO04340), the Jak-STAT (KO04630), the Notch (KO04330), the Hippo (KO04391), the FoxO (KO04068), the Ca^2+^ (KO04020) the cAMP (KO04024), the cGMP (KO04022), the PI3K-Akt (KO04151) and the Phosphatidylinositol (KO04070) pathway^12^,^48^,^52^,^53^,^54^,^55^. Components of these signaling pathways have been highly evolutionarily conserved among metazoans^56^, and are active in the testis transcriptome of *L. albus*.

From a metabolic point of view, and considering the importance of energy allocation in gonad development of sea urchins, our KEGG analysis identified the highest number of sequences in carbohydrate metabolism, followed by amino acid metabolism and lipid metabolism. This is not surprising since gonad tissues have high levels of carbohydrate, protein and lipid reserves to supply energy requirements during gonad growth and gametogenic cycles^41^. Proteins and lipids play a dominant role in cellular metabolism because of their dual roles as structural and functional elements of cell membranes^57^. However, most of the cellular energy is derived from the oxidation of carbohydrates via glycolysis and the Krebs cycle^58^. The synthesis of glycogen from glucose is the most common pathway used by metazoans to store carbohydrates and the anabolic cost of glycogen synthesis is low (0.42 J mg glycogen^−1^) when compared to the energy costs of protein synthesis (13 J mg protein^−1^)^41^. This may explain the major abundance of transcripts linked to the glycolysis pathway (KO00010), the Citrate cycle (KO00020), the pentose phosphate pathway (KO00030) and pyruvate metabolism (KO00620) in our *de novo* assembled transcriptome. Nonetheless, other carbohydrate metabolic sub-pathways, such as pentose phosphate (KO00030) and fructose and mannose metabolism (KO00051), seem also to play a biochemical role in gonad development and gametogenesis in *L. albus*. These findings support the idea that the sperm of echinoderms can uptake glucose and metabolize mannose and pentose as energy sources^14^.

### Evolutionary conservation of protein coding genes in testis of echinoderms

The basic processes of spermatogenesis and male gonad development are astonishingly similar in even very different animals, and the genes responsible of these processes are highly conserved^59^. In echinoderms, for example, functional genes involved in protein folding (e.g., heat-shock proteins, endoplasmin), spermatogenesis cell cycle (e.g., serine/threonine protein kinase, cyclin), cell signaling (e.g., calcitonin peptide-receptor), sperm motility (e.g., dyneins, intraflagellar transport proteins) and development (e.g., DEAD-box proteins, Vasa, Nanos) exhibit high evolutionary conservation among the different classes^14^. Our comparative analysis identified several of these conserved orthologous genes between sea urchins and sea stars. Shared clusters between these two groups were over-represented by proteins related to the kinesin complex and molecular functions such as microtubule motor activity and hydrolase activity. The kinesin complexes generally possess a force-generating enzymatic activity, or motor, which converts the free energy of the gamma phosphate bond of ATP into mechanical work^60^. These proteins are considered a second type of motor enzyme with a wide range of essential roles in mitotic and meiotic division during spermatogenesis, and modulating the shape and function of Sertoli cells^61^. Similarly, proteins related to microtubule motor activity are fundamental components of the dramatic cellular change that takes place during spermatogenesis. These proteins participate in several important events (e.g., nuclear elongation, cytoplasmic redistribution and reduction, and development of the flagellum) through the intricate process of cellular transformation from non-polar spermatogonia into the highly polarized, functional spermatozoa^8^,^62^. On the other hand, proteins involved in hydrolase activity such as the Ubiquitin, have been implicated in the control of mammalian gametogenesis, sperm acrosomal function and anti-polyspermy defense^63^. In sea urchins, these proteins are suggested to be responsible for acrosome reaction and sperm penetration of the vitelline envelope during fertilization^64^.

Finally, our evolutionary comparison among testis transcriptomes of sea urchins and sea starts revealed different degrees of conservation of functional genes depending on the phylogenetic relationships. For example, closely related species such as *L. albus* and *A. lixula*, shared more orthologous proteins associated to spermatogenesis and development than distantly related species (e.g., *L. albus* and *S. purpuratus*), in which most of the clusters were represented by proteins linked to metabolic process and multicellular organismal development. The slow evolutionary rate of many spermatogenesis proteins may explain their greater conservation among closely related species^65^.

## Conclusion

The gonads of sea urchins have dual functions, as the main organ of nutrient storage and also as the central core for reproduction. In this work, we identified transcripts related to these functions in male gonads of the Chilean edible sea urchin *Loxechinus albus*. Our *de novo* assembled transcriptome and its functional annotation provide important molecular resources to improve our understanding of the reproductive development and spermatogenesis of sea urchins. The identification of candidate genes related to gonadal growth and maturation, somatic cell differentiation, pigmentation, gamete generation, meiosis, sperm ejaculation, fertilization and sex determination, opens a wide range of possibilities for aquaculture applications (e.g., high-quality gonads for consumption), management and conservation (e.g., natural populations recovery) and studies about the evolution of functional and structural pathways that underlie the reproductive toolkit in marine invertebrates.

## Methods

### Ethics statement

This study was carried out in strict accordance with the recommendations in the Guide for the Care and Use of Laboratory Animals of the Comisión Nacional de Investigación Científica y Tecnológica de Chile (CONICYT). All experiments were conducted according to current Chilean law. The protocol was approved by the Committee on the Ethics of Animal Experiments of the Universidad Austral de Chile.

### Animal collection, RNA extraction and sequencing

Adult individuals of *L. albus* were collected in Los Molinos (39°40′S-73°12′W), southern Chile before the spawning season of this species (Austral winter-spring) in 2013. Sex was determined by gonad examination under a microscope for the detection of sperm and eggs. Mature animals (i.e., bright gonads filled with fully formed eggs or sperm) with similar gonad indexes (i.e., percentage of the total body weight that is made up by the gonad), were selected for further analyses. Total *L. albus* RNA was extracted from fresh gonads of eleven adult males using a commercial RiboPure^TM^Kit (AMBION). Quality and quantity of the RNA were analysed by Agilent 2100 Bioanalyzer RNA assays and evaluated by calculating the ratio of the 28S and 18S ribosomal RNA intensity peaks. Total RNA was pooled and submitted to Macrogen, Seoul, Korea (http://www.macrogen.com) for cDNA library construction, library normalization, and cDNA pyrosequencing in a 454-GS FLX Titanium System. Data quality was checked at Macrogen and provided in FASTq format.

### Assembly and annotation

The sequenced raw data was processed using the CLC Genomics Workbench software v.8.2 (CLC bio, Denmark) in a four-steps pipeline: i) removal of adapter sequences; ii) removal of all reads containing more than 5% of ambiguous nucleotides (‘N’), iii) trimming base pairs with a Phred quality score ≤ 20 from the 3′-end of each sequence; and iv) removal of reads shorter than 30 bp after trimming. High quality reads were used in two different assembly strategies, the *de novo* and the Reference Annotation Based Transcript (RABT) methods. In the first case, high quality reads were assembled following the parameters described in Cea *et al*.^23^ and default settings (mapping mode = fast, automatic bubble size = yes, minimum contig length = 100 nt, automatic word size = yes, perform scaffolding = yes, auto-detect paired distances = yes) using the CLC *de novo* assembly option. For the RABT approach, the high quality reads were mapped to the *Strongylocentrotus purpuratus* reference genome^66^ using the CLC *assembly to reference* tool. In both cases, reads that were not incorporated into any contig (i.e., singletons) were discarded and excluded from further analyses. The quality and completeness of the new draft assemblies were analysed in three ways: using the software QUAST for assembly statistics^67^, analysing the coverage of homologs in UniRef90^68^ and by mean of the core eukaryotic genes mapping approach (CEGMA)^69^. The last approach is based on a subset of 248 widely conserved eukaryotic core genes that generally lack paralogs in the eukaryotes^69^.

The best-assembled unigenes were used for BLAST searches and annotation against the UniProt (Swiss-Prot and TrEMBL) and NCBI RefSeq (nr) protein databases using the BLASTX algorithm with an e-value cutoff of 1e^−5^. Annotated unigenes were further searched for Gene Ontology (GO) terms using the Blast2GO software^70^ according to the main categories of Gene Ontology (GO; molecular functions, biological processes and cellular components)^71^. Complementary annotations were done with the InterProScan v.5 software^72^, which provides functional analysis of proteins by classifying them into families and predicting domains and important sites. The annotation results were further fine-tuned with the Annex and GO slim functions of the Blast2GO software in order to improve and summarize the functional information of the transcriptome dataset. After that, the EC (Enzyme Commission) number was assigned and parsed based on the Blast2GO results. The distribution of annotated unigenes among GO categories was mapped using the WEGO software^73^. Finally, a comparison of overall nucleotide sequence homology between the male gonad transcriptome of *L. albus* and the genome of *S. purpuratus* was completed using the Kyoto Encyclopedia of Genes and Genomes (KEGG) and its automated assignment server (KAAS)^74^.

### Comparison among male gonad transcriptomes of echinoderms

In order to explore the level of evolutionary conservation of gene/protein functions in male gonads of echinoderms, we compared the testis transcriptomes of the sea urchins *L. albus, Arbacia lixula*^32^, *Evechinus chloroticus*^34^, *S. purpuratus*^22^ and the sea stars *Patiria miniata*^36^ and *Acanthaster planci*^14^. The raw unassembled datasets of these species were downloaded from the NCBI SRA database under the accessions SRP066435, SRR1014624, SRR532121, SRR573710 and SRR1197243 respectively. The same quality control and pipelines applied for *L. albus* were implemented to obtain high-quality contigs of the additional species. Transcripts of the six species were translated and used for comparisons of orthologous clusters with the OrthoVenn software^75^ using default parameters to identify GO categories and any GO enrichment.

## Data Availability

Raw reads have been deposited on GenBank public database under the accession number SRP066399 of the bioproject PRJNA302689. In addition, the assembly file has been deposited in Dryad, DOI: 10.5061/dryad.hc7v5.

## Additional Information

**How to cite this article**: Gaitán-Espitia, J. D. *et al*. Functional insights into the testis transcriptome of the edible sea urchin *Loxechinus albus. Sci. Rep.* **6**, 36516; doi: 10.1038/srep36516 (2016).

**Publisher’s note**: Springer Nature remains neutral with regard to jurisdictional claims in published maps and institutional affiliations.

## Supplementary Material

Supplementary Information
