# Supplementary material for: Functional insights into the testis transcriptome of the edible sea urchin Loxechinus albus
Source: Sci Rep. 2016 Nov 2;6:36516. doi: 10.1038/srep36516 (PMC5090362; doi:10.1038/srep36516)
Supplement: Supplementary Information [file srep36516-s1.pdf]

Version: 06-09-2016

Running Head: Sea urchin male gonad transcriptome

Functional insights into the testis transcriptome of the edible sea urchin *Loxechinus albus*

Juan Diego Gaitán-Espitia[1,2], Roland Sánchez[1], Paulina Bruning[1] & Leyla Cardenas[1*]

[1] Instituto de Ciencias Ambientales y Evolutivas, Universidad Austral de Chile, Casilla 567 Valdivia, Chile.

[2] CSIRO Oceans & Atmosphere, GPO Box 1538, Hobart 7001, TAS, Australia

* **Corresponding author**: Leyla Cardenas. Instituto de Ciencias Ambientales y Evolutivas, Universidad Austral de Chile, Casilla 567 Valdivia, Chile. Email: leylacardenas@uach.cl

*** Data deposition**

Raw reads have been deposited on GenBank public database under the accession number SRP066399 of the bioproject PRJNA302689. In addition, the assembly file has been deposited in Dryad, DOI: 10.5061/dryad.hc7v5.

SUPPORTING INFORMATION

Supplementary Figure S1. Contig length and coverage of the *de novo* assembled transcriptome of *Loxechinus albus*.

Supplementary Figure S2. Alignment length and hits of the *de novo* assembled transcriptome of *Loxechinus albus*.

Supplementary Figure S3. Distribution of GO levels within the three main categories: biological processes, cellular components and molecular functions.

Supplementary Figure S4. Mapping and annotation of *Loxechinus albus* genes (A): histogram of number of Gene Ontology terms with a given Evidence Code; (B) histogram of the number of GO obtained form each possible Database source of annotations.

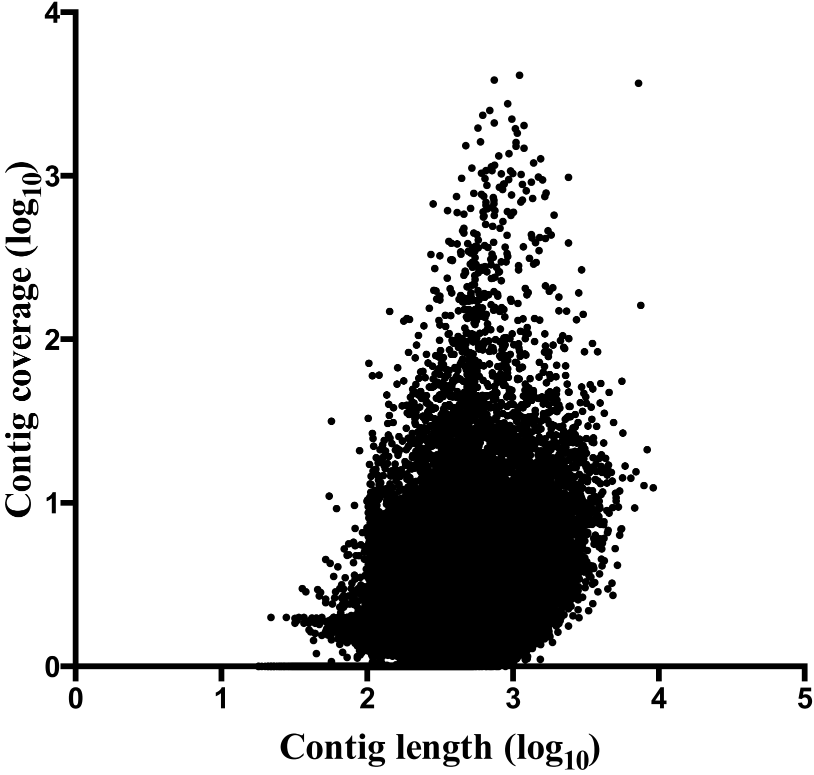
Contig coverage (log10)
Contig length (log10)
0
1
2
3
4
5

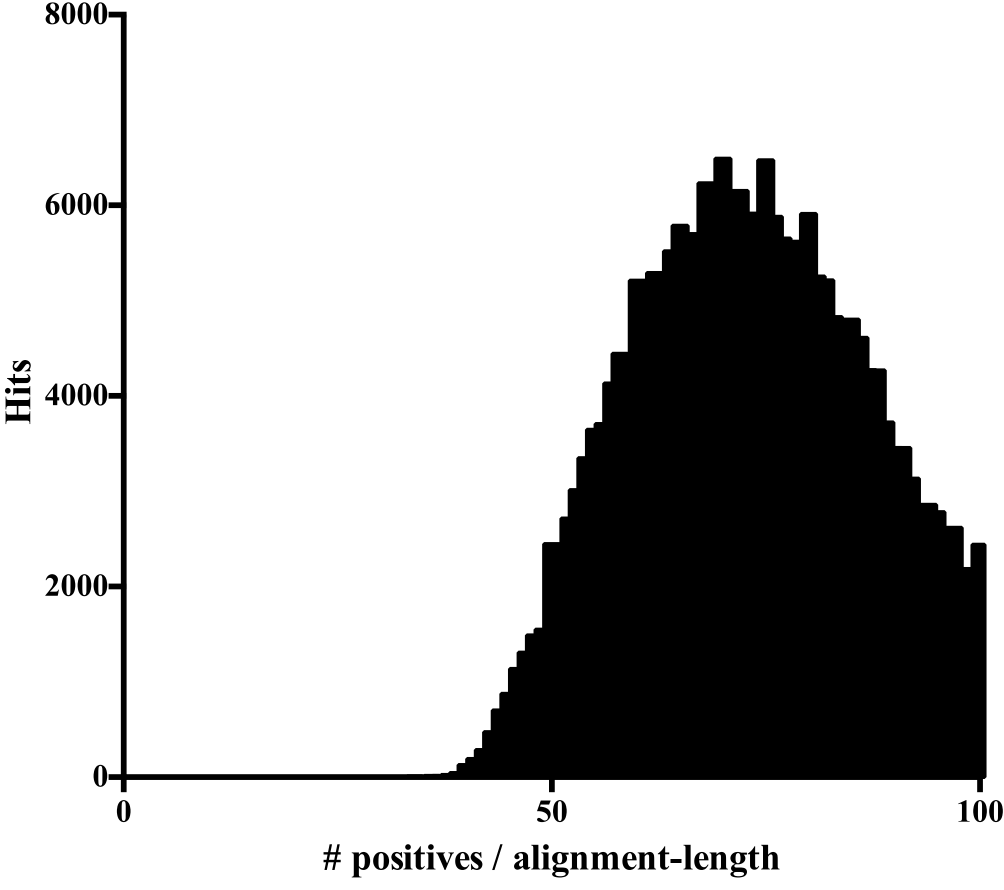

8000
6000
4000
2000
0
Hits
0
50
100
positives / alignment-length

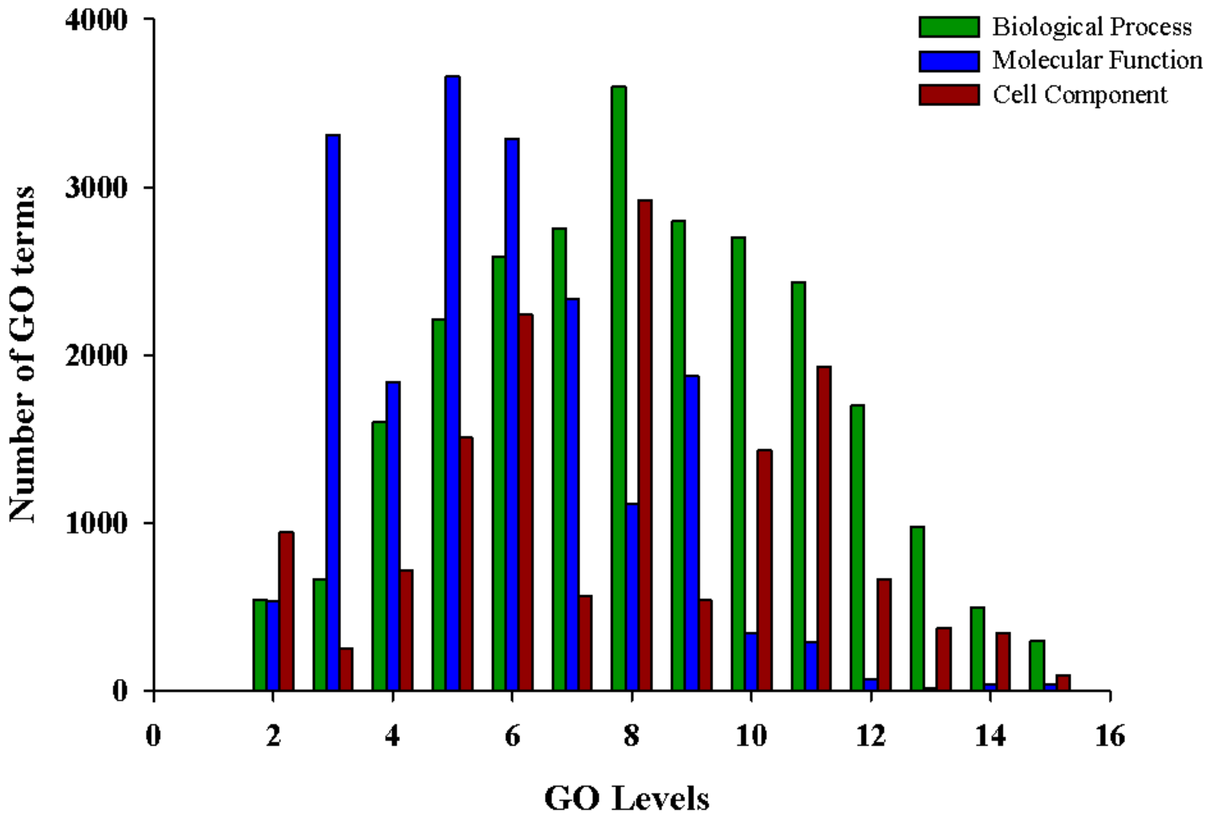

(Total Annotations = 58 957, mean level=7.26, std=2.97)

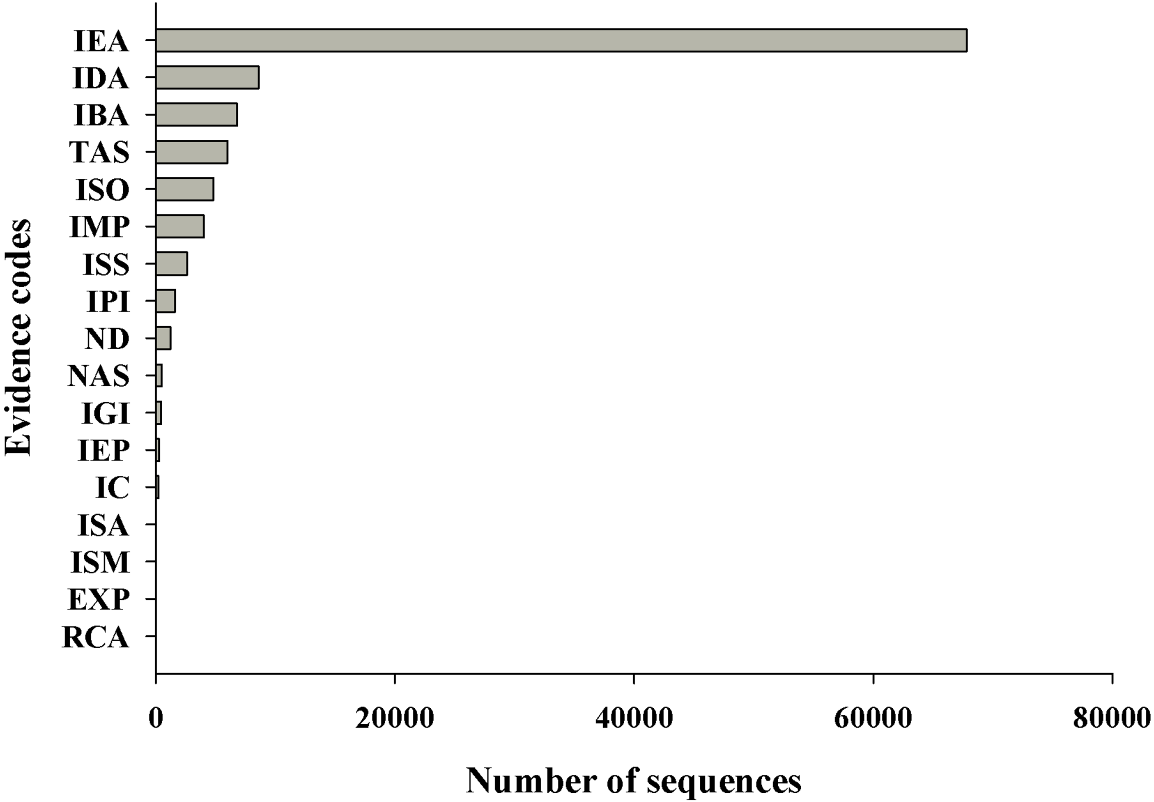
Evidence codes
IEA
IDA
IBA
TAS
ISO
IMP
ISS
IPI
ND
NAS
IGI
IEP
IC
ISA
ISM
EXP
RCA
0
20000
40000
60000
80000
Number of sequences

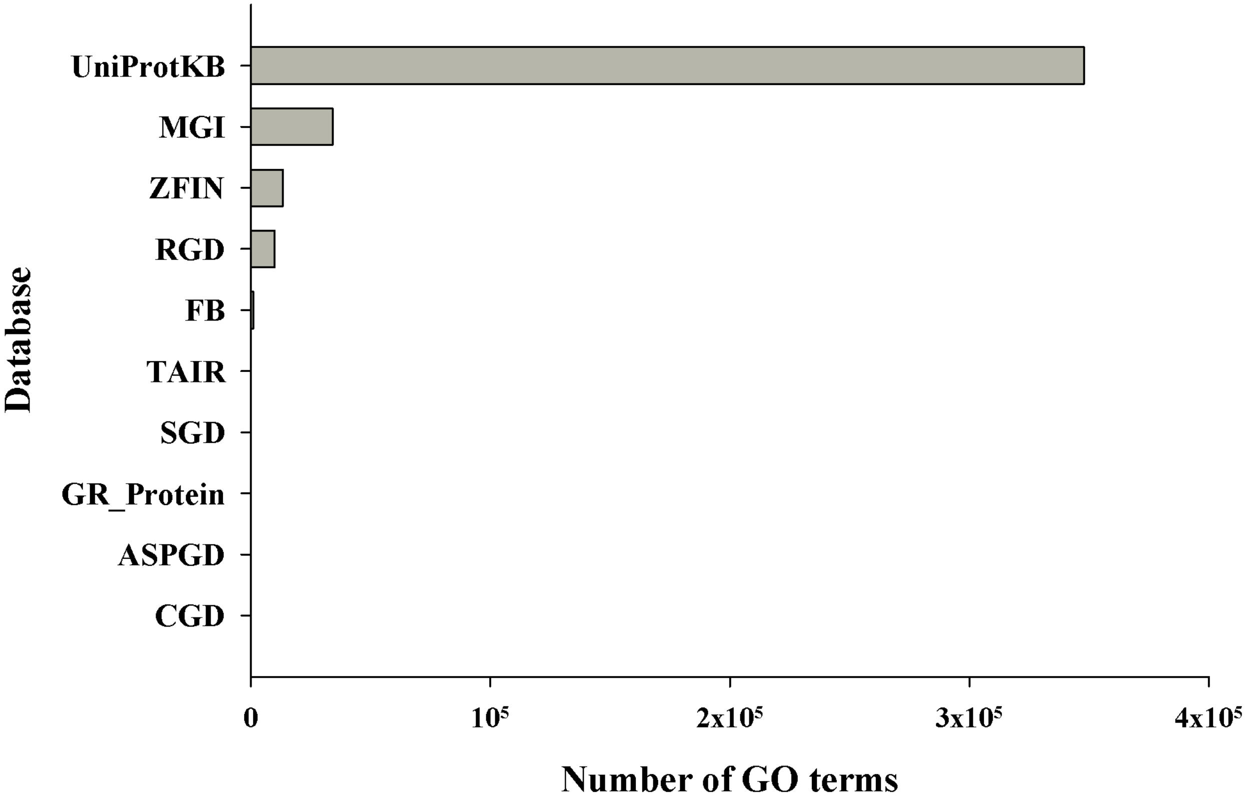

UniProtKB
MGI
ZFIN
RGD
FB
TAIR
SGD
GR_Protein
ASPGD
CGD
Database
0
10^5
2x10^5
3x10^5
4x10^5
Number of GO terms

Supplementary Table S1. Summary statistics of the transcriptome assemblies using the de novo and Reference Annotation Based Transcript (RABT) methods.

Category	de novo	RABT
Mapped reads	908 617	436 230
Mean read length (bp)	304	369
Number of isogroups	42 684	17 642
Number of isotigs	46 913	19 318
Unigene count	42 530	17 557
Mean unigene length (bp)	539	482
Total transcriptome length (bp)	23 392 852	11 801 905
Number of contigs > 1kb	4 396	3 292
N50 (bp)	645	956
L50	10 380	3 572
Singletons count	34 741	729 624
GC%	40.4	44.6
CEGMA complete (%)	71	63
CEGMA partial (%)	83	78
Average number of orthologs per CEG	2.11	2.03

Supplementary Table 2. Summary of homology blast search, mapping and annotation results of *Loxechinus albus* de novo transcriptome

Blast analysis	Number of hits
Without Blast hits	26 986
Annotated sequences from NCBI nr	15 167
Annotated sequences from Swiss-prot	10 517
Annotated sequences from UniRef90	15 072
Annotated sequences from Trembl	13 841
Annotated sequences from InterProScan	8 022
Annotated sequences from B2GO	8 144
Annotated sequences merged IPS-B2GO	11 400
Mapped GO	2 161
Final annotations after merging analyses	15 544
Total number of unigenes	42 530

Supplementary Table S3. Candidate genes involved in gonad development, reproduction and gametogenesis

Gene	GO Term	Sequence ID
Reproduction	GO:0000003	contig_20387, contig_2392, contig_7341, contig_8397, contig_24744, contig_31511, contig_34310, contig_38835, contig_42292, contig_43257,
Sexual reproduction	GO:0019953	contig_21185, contig_29161
Fertilization	GO:000956	contig_7745, contig_21632, contig_38747, contig_41448
Reproductive structure development	GO:0048608	contig_2103, contig_32824, contig_33409
Male gonad development	GO:0008584	contig_2110, contig_3907, contig_14366, contig_15263, contig_19936, contig_26838, contig_38747, contig_43081
Genitalia morphogenesis	GO:0035112	contig_4614, contig_9997, GO:0030538, contig_30722, contig_37157
Sertoli cell differentiation	GO:0060008	contig_28341
Leydig cell differentiation	GO:0033327	contig_16555
Nutrient reservoir activity	GO:0045735	contig_168, contig_279, contig_298, contig_613, contig_614, contig_943, contig_1211, contig_1439, contig_1711, contig_11048, contig_16290, contig_20080, contig_22034, contig_33025, contig_33726
Gamete generation	GO:0007276	contig_2594, contig_24968, contig_33454
Germ cell development	GO:0007281	contig_2056, contig_21187, contig_26943, contig_28341, contig_34543, contig_41447
Spermatid development	GO:0007286	contig_769, contig_1203, contig_4110, contig_6499, contig_9970, contig_10529, contig_12202, contig_14085, contig_15401, contig_1699, contig_17337, contig_21632, contig_25796, contig_41153
Sperm axoneme assembly	GO:0007288	contig_37913
Acrosome assembly	GO:0001675	contig_164, contig_5836, contig_16555
Spermatid nucleus differentiation	GO:0007289	contig_2110, contig_16555, contig_21535, contig_32718
Sperm chromatin condensation	GO:0035092	contig_999, contig_10362, contig_11194, contig_22649
Spermatid nucleus elongation	GO:0007290	contig_13921
Spermatid development	GO:0007286	contig_769, contig_1203, contig_1699, contig_4110, contig_6499, contig_9970, contig_10529, contig_12202, contig_14085, contig_15401, contig_17337,

		contig_21632, contig_25796, contig_41153
Male germ\-line stem cell division	GO:0048133	contig_4110
Spermatogenesis	GO:0007283	contig_4994, contig_42086, contig_10803, contig_10804, contig_14078, contig_16499, contig_16642, contig_16797, contig_17319, contig_17499, contig_23416, contig_24759, contig_25895, contig_26838, contig_26918, contig_27206, contig_28341, contig_2862, contig_28917, contig_29258, contig_29678, contig_30117, contig_31653, contig_31709, contig_31881, contig_31882, contig_32350, contig_33764, contig_34512, contig_35605, contig_37872, contig_38747, contig_38941, contig_38945, contig_41447, contig_41552, contig_42382, contig_42639, contig_4682, contig_7342, contig_7745, contig_7847
Regulation of meiosis	GO:0040020	contig_7375, contig_15729, contig_19011, contig_38773
Male meiosis	GO:0007140	contig_69, contig_87, contig_88, contig_525, contig_643, contig_769, contig_2594, contig_4110, contig_9247, contig_9970, contig_10276, contig_16354, contig_24759, contig_35605, contig_38941, contig_43269
Binding of sperm to zona pellucida	GO:0035036	contig_1435, contig_1610, contig_1665, contig_1998, contig_1999, contig_4051, contig_29905, contig_31821
Fusion of sperm to egg plasma membrane	GO:0007339	contig_1377, contig_2228, contig_2229, contig_4067, contig_12829
Sperm ejaculation	GO:0042713	contig_10803, contig_10804
Male courtship behavior	GO:0008049	contig_8188, contig_13974, contig_38355
Sex differentiation	GO:0007548	contig_11819, contig_12372, contig_33409
Flagellar motility	GO:0001539	contig_562, contig_619, contig_4353, contig_4428, contig_8080, contig_9399, contig_15825, contig_16560, contig_17724, contig_24267, contig_38725, contig_41867
Sperm motility	GO:0030317	contig_1275, contig_1650, contig_4110, contig_7823, contig_11709, contig_16555, contig_16767, contig_29678, contig_38945, contig_43210, contig_43211
Protein folding	GO:0006457	contig_73, contig_378, contig_469, contig_1435, contig_1492, contig_1587, contig_1610, contig_1665, contig_1719, contig_1930, contig_2175, contig_2944,

		contig_3255, contig_3751, contig_4446, contig_4620, contig_5020, contig_5750, contig_6432, contig_6549, contig_7313, contig_7375, contig_7783, contig_8255, contig_8311, contig_9522, contig_9754, contig_11323, contig_11327, contig_11377, contig_12097, contig_14519, contig_14680, contig_15865, contig_16150, contig_16217, contig_17273, contig_19665, contig_20903, "contig_22546, contig_23167, contig_26550, contig_28383, contig_34142, contig_35481, contig_35810, contig_36027, contig_37862, contig_39244, contig_39384, contig_40276
Cyclin\-dependent protein kinase	GO:0031660	contig_5594, contig_4967, contig_9970, contig_22455, contig_33869
Protein serine/threonine kinase activator	GO:0043539	contig_1731, contig_2500, contig_5414, contig_22308, contig_29540, contig_35322, contig_37465, contig_38754, contig_43257
Dynein binding	GO:0045502	contig_1305, contig_3332, contig_6430, contig_7578, contig_9587, contig_10803, contig_10804, contig_23228, contig_23833, contig_27661, contig_31694, contig_32708, contig_40094
Microtubule binding	GO:0008017	contig_10666, contig_10760, contig_12239, contig_12578, contig_13940, contig_16487, contig_16797, contig_16798, contig_17337, contig_18105, contig_18750, contig_18946, contig_19208, contig_19888, contig_19911, contig_21177, contig_22064, contig_2298, contig_2301, contig_23705, contig_24269, contig_24332, contig_25644, contig_26435, contig_26550, contig_26870, contig_2730, contig_28287, contig_28633, contig_30117, contig_30521, contig_31384, contig_31680, contig_3314, contig_33818, contig_33869, contig_34433, contig_37009, contig_37741, contig_38461, contig_39834, contig_40700, contig_40909, contig_41034, contig_41153,, contig_41827, contig_4411, contig_4781, contig_4967, contig_5083, contig_5232, contig_5423, contig_5594, contig_5745, contig_7280, contig_730, contig_7366, contig_7383, contig_7578, contig_9022
Pigment granule localization	GO:0051875	contig_220, contig_1896, contig_2566, contig_2841, contig_5020, contig_6636, contig_6755, contig_7235, contig_9650, contig_16631, contig_16842, contig_10529, contig_12029, contig_13892, contig_21909, contig_28582, contig_36855, contig_37327, contig_37465, contig_37913

Pigmentation during development	GO:0048066	contig_8822, contig_10529, contig_19586, contig_21909, contig_24305, contig_28122, contig_34967, contig_35405

Supplementary Table S4. Summary of the top protein clusters in the testis transcriptomes of six sea urchin species.

ID Cluster	Number of sequences	Swiss-Prot Hit	GO Annotation
32	12	Cell division control protein 48 homolog E	GO:0005618; GO:0005856; GO:0005829; GO:0005794; GO:0005634; GO:0005524; GO:0016787; GO:0007049; GO:0051301; GO:0015031
33	12	Kinesin-like protein FLA10	GO:0097014; GO:0005871; GO:0005874; GO:0031514; GO:0005524; GO:0003777; GO:0035720; GO:0044458
44	11	Histone deacetylase HDT3	GO:0005730; GO:0016787; GO:0046872; GO:0016568; GO:0007275; GO:0045892; GO:0009737; GO:0009651; GO:0009414; GO:0006351
67	9	Cytochrome P450 711A1	GO:0016021; GO:0020037; GO:0005506; GO:0004497; GO:0016705; GO:0009926; GO:0016117; GO:0009963; GO:0009934; GO:0010223
72	9	Cytochrome c oxidase subunit 3	GO:0016021; GO:0005743; GO:0004129; GO:0019646
74	9	Isovaleryl-CoA dehydrogenase	GO:0005739; GO:0050660; GO:0008470; GO:1902198; GO:1902196; GO:0006552
91	8	Q38953 splicing factor	GO:0005829; GO:0005739; GO:0005634; GO:0009506; GO:0005524; GO:0008026; GO:0003723; GO:0006397; GO:0008380
101	8	Protein At1g12775	GO:0005739
111	8	Early nodulin-like protein 2	GO:0031225; GO:0046658; GO:0048046; GO:0009507; GO:0005886; GO:0005773; GO:0005507; GO:0009055;
130	7	Serine/arginine-rich splicing factor SC35	GO:0005829; GO:0016020; GO:0016607; GO:0005681; GO:0000166; GO:0003723; GO:0000398; GO:0008380
135	7	Kinesin-like protein KLP1	GO:0005737; GO:0005871; GO:0005874; GO:0031514; GO:0005524; GO:0003777; GO:0030030; GO:0007018
144	7	Calnexin homolog	GO:0005783; GO:0016021; GO:0005509; GO:0030246; GO:0006457
		DNA replication licensing factor MCM3	GO:0042555; GO:0005634; GO:0005524; GO:0003677; GO:0003678; GO:0008283; GO:0000911; GO:0006306; GO:0006270; GO:0006268; GO:0016458; GO:0051567; GO:0051726; GO:0006275; GO:0009909
155	7	SNF1-related protein kinase	GO:0005524; GO:0005975; GO:0006633; GO:0042128

Supplementary Table S5. Annotation of clusters in the testis transcriptomes of six sea urchin species.

	GO Terms	Name	Counts
BP	GO:0008150	biological_process	9
	GO:0008152	metabolic process	6
	GO:0009987	cellular process	6
	GO:0044237	cellular metabolic process	5
	GO:0032502	developmental process	5
	GO:0044238	primary metabolic process	4
	GO:0006807	nitrogen compound metabolic process	4
	GO:0016043	cellular component organization	4
	GO:0032501	multicellular organismal process	4
	GO:0065007	biological regulation	4
	GO:0016070	RNA metabolic process	3
	GO:0007049	cell cycle	2
	GO:0043170	macromolecule metabolic process	2
	GO:0006996	organelle organization	2
	GO:0044255	cellular lipid metabolic process	2
	GO:0006928	cellular component movement	2
	GO:0006629	lipid metabolic process	2
	GO:0051276	chromosome organization	2
	GO:0006396	RNA processing	2
	GO:0051301	cell division	2
	GO:0006139	nucleobase-containing compound metabolic process	2
	GO:0050896	response to stimulus	2
	GO:0006082	organic acid metabolic process	2
	GO:0051641	cellular localization	1
	GO:0071555	cell wall organization	1
	GO:0043412	macromolecule modification	1
	GO:0051234	establishment of localization	1
	GO:0046483	heterocycle metabolic process	1
	GO:0051179	localization	1
	GO:0051186	cofactor metabolic process	1
	GO:0045333	cellular respiration	1
	GO:0042440	pigment metabolic process	1
	GO:0015031	protein transport	1
	GO:0006457	protein folding	1
	GO:0006464	cellular protein modification process	1
	GO:0006725	cellular aromatic compound metabolic process	1
	GO:0006304	DNA modification	1
	GO:0006260	DNA replication	1
	GO:0006091	generation of precursor metabolites and energy	1
	GO:0006259	DNA metabolic process	1
	GO:0006793	phosphorus metabolic process	1
	GO:0006810	transport	1

	GO:0016458	gene silencing	1
	GO:0022607	cellular component assembly	1
	GO:0032392	DNA geometric change	1
	GO:0005975	carbohydrate metabolic process	1
	GO:0009914	hormone transport	1
	GO:0007154	cell communication	1
	GO:0008283	cell proliferation	1
	GO:0032989	cellular component morphogenesis	1
MF	GO:0043167	ion binding	12
	GO:0000166	nucleotide binding	8
	GO:0001882	nucleoside binding	6
	GO:0005488	binding	6
	GO:0016787	hydrolase activity	4
	GO:0003676	nucleic acid binding	3
	GO:0016491	oxidoreductase activity	2
	GO:0009055	electron carrier activity	2
	GO:0003774	motor activity	2
	GO:0004386	helicase activity	2
	GO:0046906	tetrapyrrole binding	1
	GO:0048037	cofactor binding	1
	GO:0030246	carbohydrate binding	1
	GO:0030234	enzyme regulator activity	1
	GO:0004497	monooxygenase activity	1
CC	GO:0044464	cell part	8
	GO:0005575	cellular_component	7
	GO:0005622	intracellular	7
	GO:0016020	membrane	6
	GO:0005634	nucleus	5
	GO:0005739	mitochondrion	4
	GO:0043226	organelle	4
	GO:0043229	intracellular organelle	4
	GO:0005856	cytoskeleton	3
	GO:0043234	protein complex	3
	GO:0005929	cilium	2
	GO:0005618	cell wall	2
	GO:0042995	cell projection	1
	GO:0055044	symplast	1
	GO:0005794	Golgi apparatus	1
	GO:0005681	spliceosomal complex	1
	GO:0005576	extracellular region	1
	GO:0005730	nucleolus	1
	GO:0005773	vacuole	1
	GO:0005783	endoplasmic reticulum	1
	GO:0009536	plastid	1

Running Head: Sea urchin male gonad transcriptome

Tasting the sea: Functional insights into the testis transcriptome of the edible sea urchin *Loxechinus albus*

Juan Diego Gaitán-Espitia[1,2], Roland Sánchez[1], Paulina Bruning[1] & Leyla Cardenas[1*]

[1] Instituto de Ciencias Ambientales y Evolutivas, Universidad Austral de Chile, Casilla 567 Valdivia, Chile.

[2] CSIRO Oceans & Atmosphere, GPO Box 1538, Hobart 7001, TAS, Australia

* **Corresponding author**: Leyla Cardenas. Instituto de Ciencias Ambientales y Evolutivas, Universidad Austral de Chile, Casilla 567 Valdivia, Chile. Email: leylacardenas@uach.cl

*** Data deposition**

Raw reads have been deposited on GenBank public database under the accession number SRP066399 of the bioproject PRJNA302689. In addition, the assembly file has been deposited in Dryad, DOI: 10.5061/dryad.hc7v5.
